# Arterial phase enhancement of the adrenal glands as a severity indicator for acute pancreatitis

**DOI:** 10.1007/s11604-025-01752-2

**Published:** 2025-02-18

**Authors:** Ryusuke Ookura, Noriaki Usuki, Yukio Miki

**Affiliations:** 1 Department of Diagnostic and Interventional Radiology, Japan Community Healthcare Organization Osaka Hospital, 4-2-78, Fukushima, Fukushima-ku, Osaka, 553-0003 Japan; 2 Department of Diagnostic and Interventional Radiology, Graduate School of Medicine, Osaka Metropolitan University, 1-4-3, Asahi-Machi, Abeno-Ku, Osaka, 545-8585 Japan

**Keywords:** Acute pancreatitis, Adrenal gland, Dynamic contrast-enhanced computed tomography, Intense adrenal enhancement

## Abstract

**Purpose:**

To investigate the relationship between the computed tomography (CT) value of the adrenal glands in the arterial phase of dynamic contrast-enhanced CT and the severity of acute pancreatitis.

**Materials and methods:**

We measured the maximum CT values of the adrenal glands on the arterial phase of dynamic contrast-enhanced CT of patients with acute pancreatitis and compared them with those of the non-pancreatitis group. Moreover, we evaluated the correlations between the adrenal CT values and maximum C-reactive protein (CRPmax) and CRP/albumin ratio (CAR) in the clinical courses.

**Results:**

In this retrospective study, a total of 68 patients was included. The maximum CT value of the adrenal glands of pancreatitis group was significantly higher than that of the control group (p < 0.001). Significant fair correlations were observed between the adrenal CT value and CRPmax (r = 0.483, p < 0.001) or CAR (r = 0.450, p < 0.001). The cut-off value of the adrenal CT values was determined as 180.5 Hounsfield unit.

**Conclusion:**

In cases of acute pancreatitis, the maximum CT value of the adrenal gland in the arterial phase of dynamic contrast-enhanced CT was significantly higher than in non-pancreatitis controls, and the intensity of the contrast enhancement correlated with the CRPmax and CAR during the subsequent course of pancreatitis. This finding may assist in predicting the severity of acute pancreatitis.

## Introduction

The adrenal glands are sometimes strongly enhanced from the arterial to portal phase on dynamic contrast-enhanced computed tomography (CT) in cases of trauma-induced hypovolemic shock. Kaufman was the first to mention this finding [1], followed by reports that this strong contrast enhancement was associated with a poor prognosis [2, 3]. This finding has also been reported for patients admitted to the intensive care unit (ICU) [4, 5]. Recently, the finding that only the adrenal cortex is strongly enhanced and the adrenal medulla is poorly enhanced in septic shock has been reported. This finding is called “hollow adrenal gland sign (HAGS) and suggests a poor prognosis [6, 7]. It also reported that a larger standard deviation of CT values of the adrenal glands is associated with a poorer prognosis [8]. Additionally, in cases without shock, an increased enhancement of the adrenal glands has been reported in the portal venous phase of contrast-enhanced CT of acute mesenteric ischemia [9].

Although not a case–control study, Bollen et al. reported three cases of acute pancreatitis with similar findings [10]. The cases of mild acute pancreatitis in which inflammation is restricted to the pancreas and its surroundings have a relatively good prognosis, whereas severe cases with the involvement of other organs can be fatal. Therefore, many indicators have been developed to predict the severity of pancreatitis at the earlier stage after the onset. As simple indicators using general blood tests, for example, Wilson et al. [11] and Stirling et al. [12] reported serum CRP at 210 mg/L and 190 mg/L as cut-off values for severe pancreatitis, respectively. Recently, some studies have reported that the CRP/albumin ratio (CAR) reflects prognosis better than CRP [13]. For example, Yılmaz et al. [14] reported 8.51 and Haider Kazmi et al. [15] reported 4.35 as the cut-off values of CAR for severe pancreatitis. In addition, many methods of assessing severity using imaging have been created, and the Balthazar score [16] and its modified version, the modified CT severity index (MCTSI) [17], are widely used.

Based on the above, we hypothesized that the intensity of contrast enhancement of the adrenal glands might be one of the indicators to predict the severity and prognosis of acute pancreatitis. Therefore, we investigated the intensity of contrast enhancement of the adrenal glands in the arterial phase of dynamic contrast-enhanced CT in cases of acute pancreatitis. We then examined the association between the intensity of contrast enhancement and the severity of pancreatitis.

## Methods

### Study participants

The ethics committee of our hospital approved the use of clinical data and the retrospective study design (#2024–025, Oct 29, 2024). This study was carried out by the Declaration of Helsinki.

While preparing this manuscript, the authors used natural language processing software (DeepL and Grammarly) for Japanese-to-English translation and English proofreading and modified the sentences as needed.

All patients who had undergone dynamic contrast-enhanced CT of the upper abdomen at presentation or within one week after admission and were finally diagnosed with acute pancreatitis at our hospital between August 2021 and July 2024 were included in the pancreatitis group. We excluded the patients who were not hospitalized, presented more than one week after the onset of pancreatitis, or were complicated with infection. For patients who underwent multiple CT scans during the study period, we used only the earliest one and excluded the others.

Patients who had undergone dynamic contrast-enhanced CT for examination of benign and chronic lesions such as pancreatic cystic lesions and hepatic hemangiomas within the study period were randomly selected by matching age and gender, and these were used as the control group. Cases with malignant tumors, pancreatitis/pseudocysts, liver cirrhosis, and massive ascites were excluded.

### CT acquisition and imaging analysis

Abdominal CT was performed using 64- or 80-detector-row scanners (Aquilion, Toshiba Medical Systems, Tokyo, Japan). Images were acquired with 0.5–0.75 s rotetaion time, 1.0- (64-detector-row scanner) or 0.5- (80-detector-row scanner) mm beam collimation, pitch factor of 0.8, tube voltage of 120 kV, and 32–36 cm scanning field of view. Weight adopted warmed iopamidol (Iopamiron 370^®^, Bayer Pharma Japan, Osaka, Japan) or iohexol (Omnipaque 300^®^, GE Healthcare Japan, Tokyo, Japan) were used as the intravenous contrast agent with a flow rate of 3–4 ml/s through a 22-gauge catheter in an antecubital vein using a mechanical power injector.

**Fig. 1:**
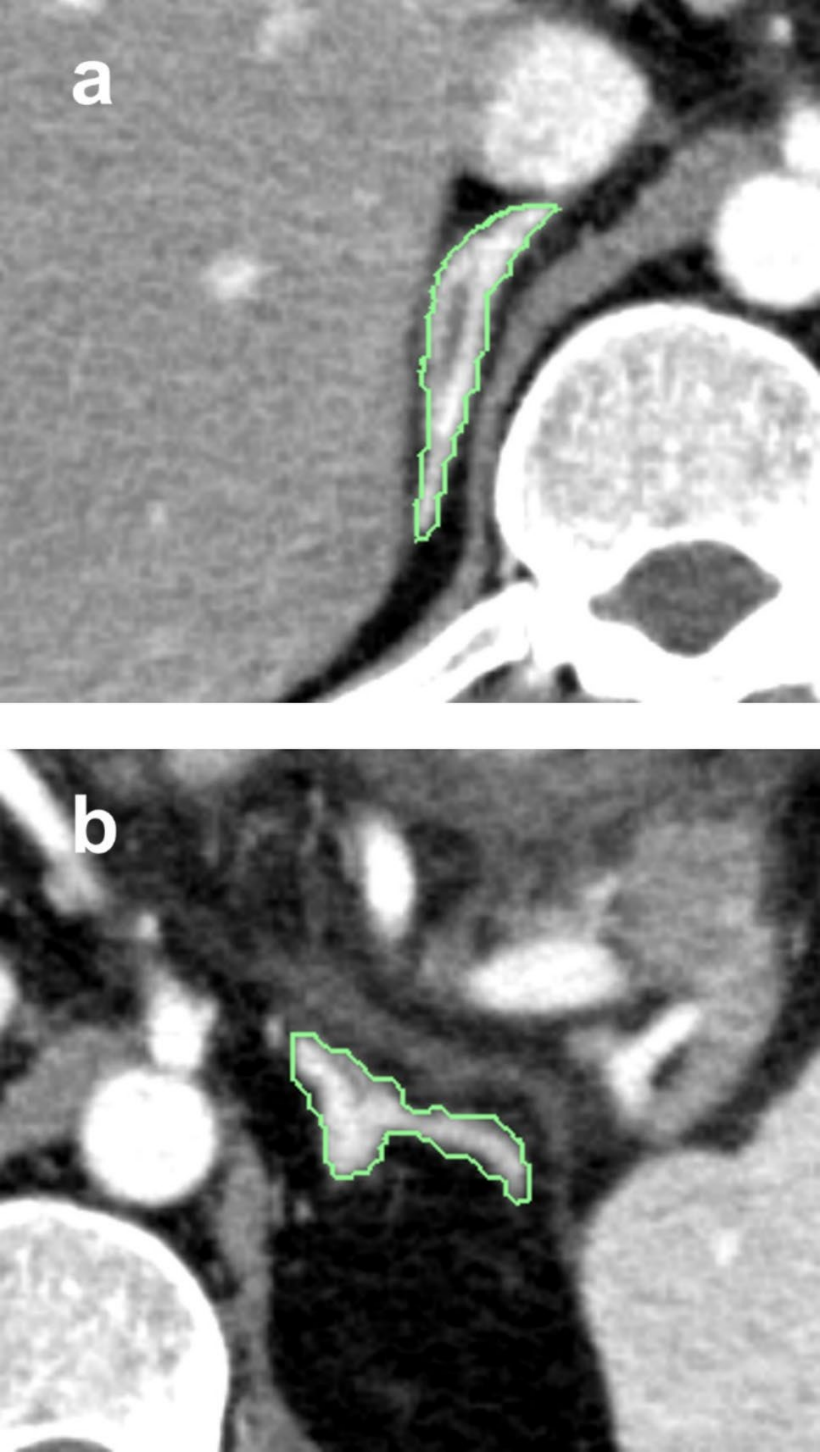
Assignment of region of interest on the bilateral adrenal glands. **a** right adrenal gland, **b** left adrenal gland

We used the 1-mm slice images of the arterial phase (40 s after contrast injection) of abdominal dynamic contrast-enhanced CT for the analysis. Avoiding visible adrenal vessels and adrenal adenomas, three slices that approximately equally divided each right and left adrenal gland into four sections between the cranial and caudal edges were selected. Because the adrenal gland is small and has a complex morphology, measuring average CT values by placing regions of interest (ROI) inside it, like for the liver, is complicated and inaccurate. Therefore, for simplicity and accuracy of measurement, we enclosed the entire adrenal glands in a ROI and used the internal maximum CT values. A board-certificated radiologist with 10 years of diagnostic imaging experience placed a freehand ROI surrounding the bilateral adrenal glands, avoiding adjacent non-fat tissue (especially the contrast-enhanced blood vessels) on the reading system (Synapse SAI viewer, Fujifilm Medical Co. Ltd., Tokyo, Japan), without reference to clinical information (Fig. 1). The maximum CT values in the ROI were measured for three slices each on the right and left adrenal glands. Their medians were taken as the right and left adrenal CT values, respectively, and the average of the medians was calculated as the index of adrenal enhancement (AdrE). AdrE was measured for the pancreatitis and control groups, respectively, and it was investigated whether the difference was significant or not. The severity of pancreatitis was also assessed using MCTSI [17] for individual CT images of the pancreatitis group.

We compared the AdrE of the pancreatitis and control groups. Furthermore, we stratified the cases by age and investigated the differences in AdrE by aging.

### Association of the contrast enhancement of the adrenal glands with maximum CRP and CAR

Maximum values during the clinical course of pancreatitis were collected for serum amylase (AMYmax, IU/L) and CRP (CRPmax, mg/L) from blood test data. Serum albumin levels (Alb) on the same day as CRPmax were also collected and CAR (= CRPmax/Alb) was calculated. The correlation between AdrE and each of AMYmax, CRPmax, and CAR was investigated.

Then, we determined the cut-off values of CRPmax and CAR that would maximize the area under the curve (AUC) of the receiver operating characteristic curve (ROC curve) when dividing the cases into two groups by the cut-off values. First, for CRP, the cut-off values for severe pancreatitis were reported, for example, 210 mg/L based on Ranson criteria by Wilson et al. [11] and 190 mg/L based on Atlanta classification by Stirling et al. [12]. Therefore, we determined the ROC of AdrE for each of the two groups by changing the boundary of the two groups between 180 and 230 mg/L in 10 mg/L increments. Similarly, for CAR, the cut-off values for severe pancreatitis were reported, for example, 8.51 based on Ranson criteria by Yılmaz et al. [14] and 4.35 based on Balthazar score by Haider Kazmi et al. [15]. This suggested that the cut-off value should be between 3.0 and 10.0. Therefore, we determined the ROC curve of the AdrE with the boundary of the two groups varying between 3.0 and 10.0 in increments of 1.0. The ROC curves with the maximum AUC were determined for each of CRPmax and CAR, and the cut-off values were obtained.

### Statistical analysis

Statistical analysis was performed using EZR software (version 1.50; Saitama Medical Center, Jichi Medical University, Saitama, Japan) [18]. The equal variances of the data were evaluated by the F test and the normalities by the Kolmogorov–Smirnov test to determine the test method to be used. Student’s or Welch’s t-test was used to compare the two groups. Paired t-test was used to compare the right and left adrenal CT values. ANOVA followed by Tukey and Bonferroni post hoc test or the Kruskal–Wallis test followed by Steel–Dwass and Holm post hoc test was used to compare AdrE according to the severity of MCTSI and according to age group. Spearman’s correlation coefficient was used to evaluate the correlation. We interpreted the correlation coefficient as follows: r < 0.3, poor; 0.3–0.6, fair; 0.6–0.8, moderate; and ≥ 0.8, very strong [19]. Statistical significance was set at p < 0.05. The cut-off values of AdrE for CRPmax and CAR were determined by the Youden index.

## Results

There were 80 examinations diagnosed as acute pancreatitis during the study period. Two patients who were not hospitalized, one patient who presented more than one week after the onset of pancreatitis, and one patient with pancreatitis due to pseudocyst infection were excluded. For six patients who underwent CT scans twice and one patient who underwent three times due to repetitive acute pancreatitis during the study period, we used only the earliest one and excluded the other eight. A total of 68 patients were included in this study. The patient profiles are shown in Table 1. In the pancreatitis group, there were four cases of death. The most common cause of pancreatitis was alcoholism, followed by cholelithiasis. The dynamic contrast-enhanced CT was performed on average 1.4 ± 1.4 days after the onset of the disease.

**Table 1:** Overview of patient profile

	Acute pancreatitis group (N = 68)	Control group (N = 68)	p value
Age (years, mean ± SD)^†^	58 ± 17	59 ± 18	0.731
Sex (male/female)	43/25	43/25	–
Cause of the pancreatitis			
Alcohol	32 (47.1%)	–	–
Cholecystolithiasis	14 (20.6%)	–	–
Hypertriglyceridemia	2 (2.9%)	–	–
Pancreatic cancer	2 (2.9%)	–	–
ERCP related	3 (4.4%)	–	–
Idiopathic	11 (16.2%)	–	–
Others	4 (5.9%)	–	–
MCTSI			
Mild	21 (30.9%)	–	–
Moderate	42 (61.8%)	–	–
Severe	5 (7.4%)	–	–

*ERCP* endoscopic retrograde cholangiopancreatography, *MCTSI* modified CT severity index. †Student’s t-test

**Fig. 2:**
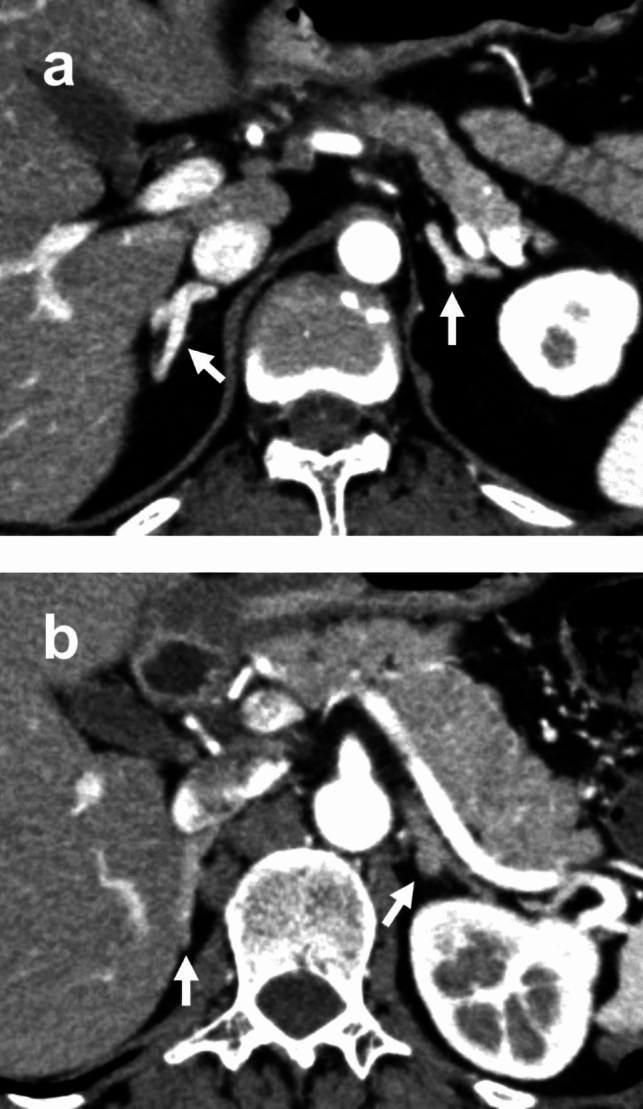
Arterial phase enhancement of the adrenal glands (arrow) of a 55-year-old female of the pancreatitis group (**a**) and a 55-year-old female of the control group (**b**). Strong enhancement of the bilateral adrenal glands was observed only in the patient with pancreatitis. MCTSI score of this pancreatitis case was 2 points (mild)

The representative images of the enhanced adrenal glands of pancreatitis and control group were shown in Fig. 2. The AdrE in the pancreatitis and control groups are shown in Table 2. CT values of both the right and left adrenal glands in the pancreatitis group were significantly higher than that in the control group. In addition, when the right and left adrenal glands were compared, the maximum CT value of the left adrenal gland was significantly higher than that of the right one in both the pancreatitis and control groups. There were no gender differences in AdrE in either the pancreatitis group or the control group.

**Table 2:** Difference of AdrE between pancreatitis and control group

Adrenal CT value (HU)	Acute pancreatitis group (N = 68)	Control group (N = 68)	p value (pancreatitis vs control)
Right adrenal CT value^†^	176.9 ± 26.8	156.4 ± 22.3	< 0.001*
Left adrenal CT value^‡^	184.5 ± 30.8	162.1 ± 21.6	< 0.001*
Mean of the right and left adrenal CT value (AdrE)^‡^	180.7 ± 27.7	159.2 ± 20.4	< 0.001*
p value (right vs left)^††^	< 0.001*	0.004*	

*AdrE* index of adrenal enhancement, *HU* Hounsfield unit. All data were presented as mean ± SD. †Student’s t-test, ‡Welch’s t-test, ††paired t-test. *p < 0.05

**Fig. 3:**
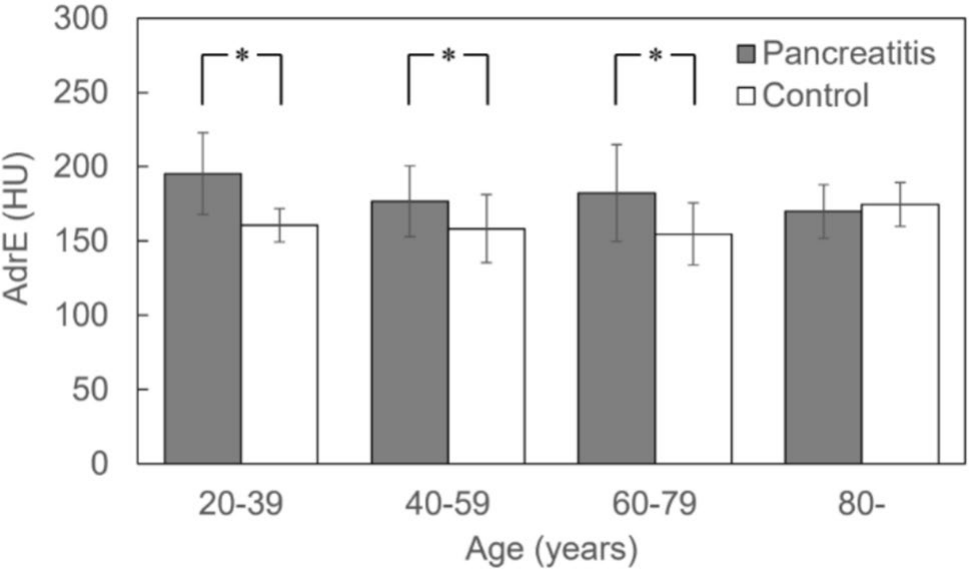
The difference in AdrE between patients with and without pancreatitis. The number of patients: 11 cases of 20–39 years, 26 cases of 40–59 years, 23 cases of 60–79 years, and 8 cases of 80 years or older in both groups. Welch’s t-test for 20–39 years age group and Student’s t-test for other age groups. *: p < 0.05

Figure 3 shows AdrE when the cases were stratified by age in 20-year intervals. The number of patients in each group is 11 cases of 20–39 years, 26 cases of 40–59 years, 23 cases of 60–79 years, and 8 cases of 80 years or older in both groups. In all age groups except those over 80, the pancreatitis group had significantly higher AdrE than the control group, but the significant difference disappeared in those over 80.

Table 3 shows the number of cases of each severity in MCTSI and their AdrE. There were no significant differences in AdrE and AMYmax between each severity of MCTSI (p = 0.309 and 0.978, respectively, by one-way ANOVA). On the other hand, CRPmax (p < 0.001) and CAR (p = 0.005) showed significant differences by ANOVA and Kruskal–Wallis test, respectively. In Tukey and Bonferroni post hoc tests, CRPmax showed significant differences between mild and severe (p < 0.001 for both post hoc tests) and moderate and severe (p < 0.001 for both post hoc tests). Also, in Steel–Dwass and Holm post hoc test, CAR showed significant differences between mild and severe (p = 0.002 and p < 0.001, respectively) and moderate and severe (p = 0.024 and p = 0.012, respectively).

**Table 3:** Differences in each severity parameter between the severity grades of MCTSI

	MCTSI			p value
	Mild	Moderate	Severe	
AdrE (HU)	173.3 ± 21.9	183.4 ± 28.7	189.3 ± 40.2	0.309
AMYmax (IU/L)	1117.3 ± 1347.8	1186.0 ± 1319.4	1067.2 ± 1993.2	0.978
CRPmax (mg/L)	124.7 ± 70.6	170.3 ± 114.2	356.1 ± 73.6	< 0.001*
CAR	4.1 ± 2.3	6.2 ± 4.9	13.5 ± 4.0	0.005*

*MCTSI* modified CT severity index, *AdrE* index of adrenal enhancement, *HU* Hounsfield unit, *AMY* amylase, *CRP* C-reactive protein, *CAR* CRP/albumin ratio. All data were presented as mean ± SD. *p < 0.05

**Fig. 4:**
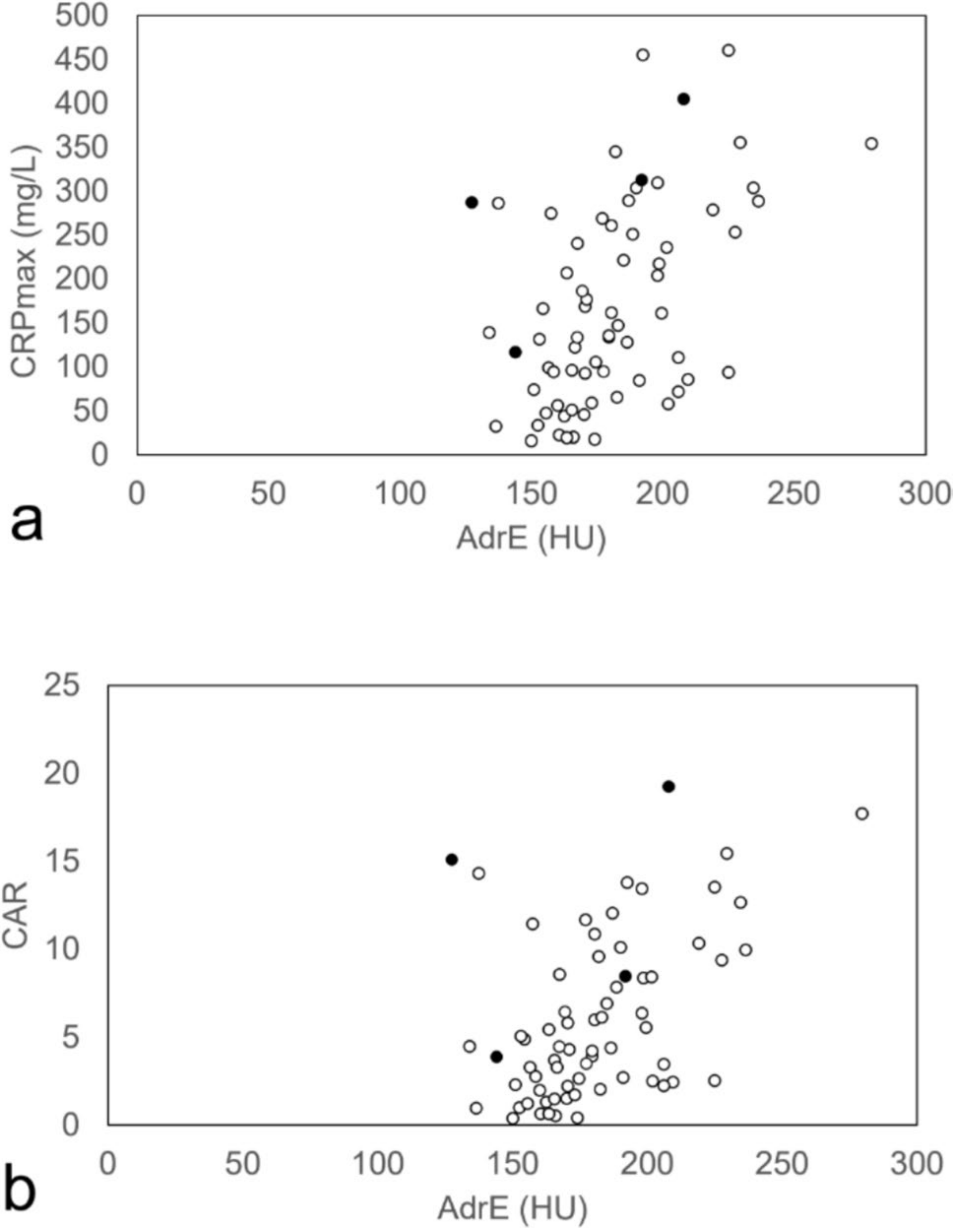
All patients plotted by CRPmax (**a**) and CAR (**b**). Spearman’s correlation coefficient with AdrE was 0.483 (p < 0.001) for CRPmax and 0.450 (p < 0.001) for CAR. Black circles are the patients who died during the study period

CRP reached the CRPmax during the clinical course at a mean of 3.4 ± 1.7 days after the onset. Scatter plots of AdrE versus CRPmax and CAR for all patients in the pancreatitis group are shown in Fig. 4. Spearman’s correlation coefficient with AdrE was 0.483 for CRPmax and 0.450 for CAR (both p < 0.001). Of the four fatal cases, three had an MCTSI score of 4, and one had an MCTSI score of 0. None of the cases were determined to be severe on CT at the admission. The AdrE of the four fatal cases were 127.5 Hounsfield unit (HU), 144 HU, 192 HU, and 208 HU; CRPmax was 286.8 mg/L, 116.1 mg/L, 312.2 mg/L, and 404.4 mg/L; CAR was 15.10, 3.87, 8.44, and 19.26, respectively.

**Fig. 5:**
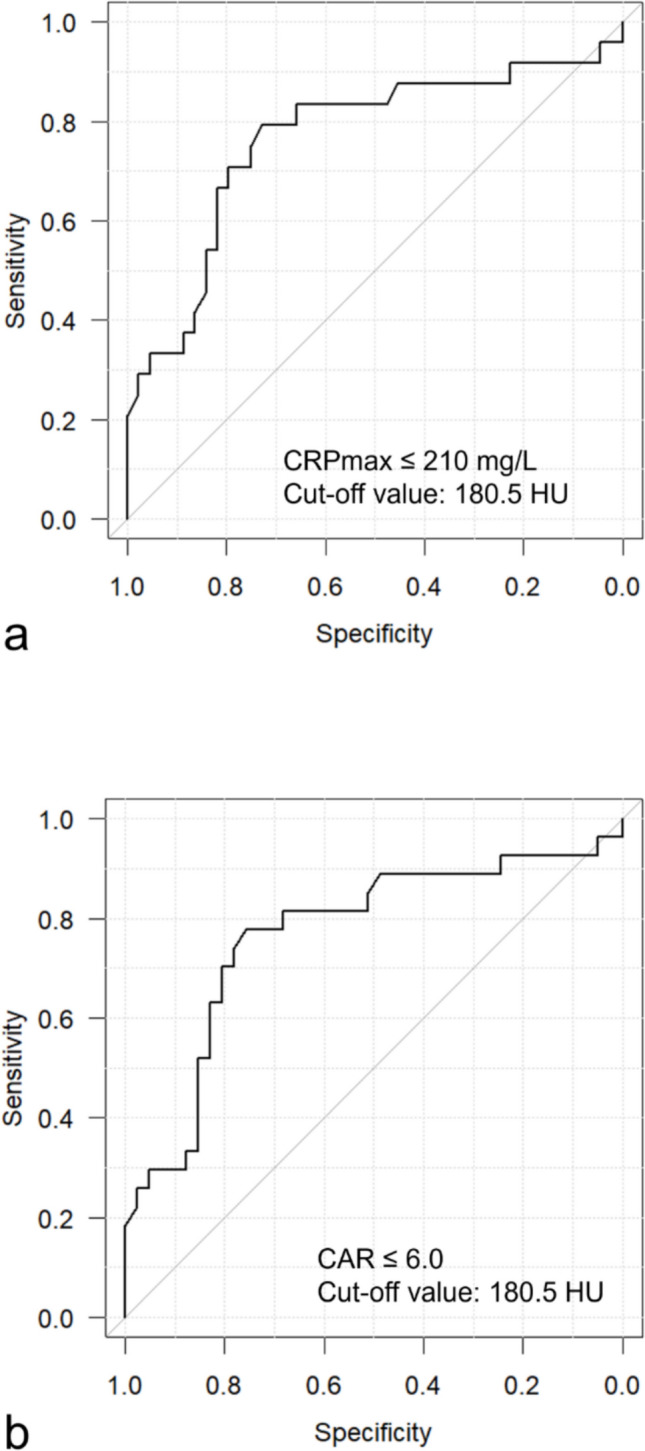
ROC analysis to predict CRPmax of 210 mg/L or more (**a**) and CAR of 6.0 or more (**b**). AUCs were 0.772 for CRPmax and 0.775 for CAR. The cut-off values are 180.5 HU for both CRPmax and CAR

When the cases were divided into two groups by CRPmax under and over 210 mg/L, the AUC had a maximum value of 0.764. In this case, the cut-off value of AdrE was calculated as 180.5 HU (Fig. 5a). Similarly, when the cases were divided into two groups by CAR under and over 6.0, the AUC had a maximum value of 0.766 and the cut-off value of AdrE was also 180.5 HU (Fig. 5b).

Based on the above, when the maximum CT value of the adrenal gland was 180.5 HU or higher, the sensitivity for CRPmax of 210 mg/L or more was calculated to be 79.2%, specificity 72.7%, positive predictive value 61.3%, and negative predictive value 86.5%. Also, the sensitivity for CAR of 6.0 or more was calculated to be 77.8%, specificity 75.6%, positive predictive value 67.7%, and negative predictive value 83.8%.

In addition, regarding the relationship with MCTSI, 8 of 21 patients (38.1%) evaluated as mild, 19 of 42 patients (45.2%) as moderate, and 4 of 5 patients (80.0%) as severe had AdrE of 180.5 HU or higher. Especially for the cases evaluated as moderate, when CRPmax and CAR were compared between the two groups of patients with AdrE less than 180.5 HU and 180.5 HU or more, the former group had CRPmax 108.0 ± 80.8 mg/L and CAR 3.81 ± 3.75, whereas the latter group had CRPmax 245.7 ± 103.8 mg/L and CAR 9.20 ± 4.44, both significantly higher in the latter (p < 0.001).

## Discussion

The results of this study indicated that the contrast enhancement of the adrenal glands increased in the arterial phase of dynamic contrast-enhanced CT in acute pancreatitis. This strong enhancement correlated with CRP and CAR during the following course of pancreatitis. In addition, if the adrenal CT values on the arterial phase of dynamic contrast-enhanced CT at the time of diagnosis of acute pancreatitis were greater than 180.5 HU, it was estimated that the patient was more likely to have CRP greater than 210 mg/L and CAR greater than 6 g/L during the following clinical course.

As mentioned above, increased contrast enhancement of the adrenal glands was originally reported in hypovolemic shock, followed by septic shock, and then by acute mesenteric infarction. In hypovolemic shock, the sympathetic nervous system responds to decreased blood pressure and maintains blood flow to the vital adrenal glands [2]. In addition, acute inflammation increases cortisol secretion due to increased adrenal function by activating the hypothalamus–pituitary–adrenal axis [20], which may result in a subsequent increase in blood flow to the adrenal glands in septic shock. In acute pancreatitis, both a hypovolemic state due to extravascular leakage of water caused by increased vascular permeability associated with inflammation and a septic state due to complications of infection can occur, and it is assumed that the contrast enhancement of the adrenal glands is likely to be increased even if the patient has not reached shock. However, since the contrast enhancement of the adrenal gland can also be observed in other pathological conditions, it would be difficult to differentiate acute pancreatitis from others by this finding.

In this study, dynamic contrast-enhanced CT was taken at the time of presentation, i.e., 0–2 days after onset in most cases, while CRPmax was usually seen 3–4 days after the onset. Previous studies have also shown that CRP in acute pancreatitis peaks not at the time of presentation but 36–72 h later [21]. At the time of CT imaging, it is usually unclear how the inflammation will exacerbate thereafter, which has led to the development of various methods for severity assessment. The results of this study suggest that the strong contrast enhancement of the adrenal glands may assist in the prediction of severity. Only a few fatal or severe cases classified as severe by MCTSI were included in this study, however, some cases were classified as moderate by MCTSI even if their CRP and CAR suggested that they were also severe. Increased CT value of the adrenal glands in the arterial phase of dynamic contrast-enhanced CT, which is usually undergone for the diagnosis of acute pancreatitis, may help detect the moderate cases closer to severe that are indistinguishable by MCTSI.

In addition, there was no strong contrast enhancement of the adrenal glands in the age group older than 80. The hypothalamus–pituitary–adrenal axis changes its activity with aging, with an increase in the evening to midnight cortisol peak, a decrease in the amplitude of the circadian cycle, and a decrease in the negative feedback of cortisol to the hypothalamus [22, 23]. On the other hand, some studies have shown no age-related changes in cortisol peaks in acute myocardial infarction or adrenocorticotropic hormone load [24], so there are many unknowns. Moreover, it has been reported that stress-responsive adrenaline secretion from the adrenal glands decreases despite the increase in activity of the sympathetic nervous system according to age [23, 25]. Such age-related changes in adrenal function, particularly in stress response, may also contribute to the intensity of the enhancement of the adrenal glands in acute pancreatitis. Further studies are needed.

In this study, we used the average intensity of the contrast enhancement of the right and left adrenal glands. However, as shown in Table 2, the intensity of the contrast enhancement of the right and left adrenal glands was significantly different, and the left side was more strongly enhanced than the right side in both pancreatitis and control groups. This may be because the left adrenal gland is generally larger than the right one [26], and the arteriovenous diameter distributed to the adrenal gland may be also larger, which may have affected the maximum CT value of the adrenal gland. Besides we could evaluate the bilateral adrenal glands in all patients in both pancreatitis and control groups in this study, this difference should be considered in such cases as one adrenal gland is atrophic or post-adrenalectomy.

There are several limitations associated with the present study. First, because this was a single-centered retrospective study, and almost all patients were Japanese, some selection biases may have been present. Second, because only a few fatal cases were included in this study, we could not examine the relationship between the adrenal CT value and mortality. Third, we examined the intensity of the contrast enhancement but did not the morphological pattern. Although some cases showed contrast enhancement patterns like HAGS or strong and heterogeneous enhancement, we could not suggest an objective method to evaluate these findings, so we did not deal with them in this study.

On the other hand, the advantages of our method include the possibility of predicting the subsequent course of acute pancreatitis, which is evaluated as moderate on contrast-enhanced CT at the presentation; unlike many previous studies using mean CT values of the adrenal glands, we use maximal CT values as a index of adrenal contrast enhancement, which allows us to ignore the influence of fat included in the ROI; the use of the median value after evaluating the maximum CT value of the adrenal glands in three slices reduces the influence of adjacent fat, partial volume effects, and internal adrenal vessels and calcifications that can become outliers; and the method can be easily applied to other acute diseases for which dynamic contrast-enhanced CT is usually performed.

As described above, we may predict the prognosis of acute pancreatitis by evaluating the contrast enhancement of the adrenal gland in the arterial phase of dynamic contrast-enhanced CT. Especially in cases of acute pancreatitis judged as moderate by MCTSI, if the adrenal gland is strongly enhanced in the arterial phase, the risk of subsequent deterioration may be higher than the other cases. In such cases, early admission to the intensive care unit and aggressive therapeutic intervention may reduce the mortality, complications, and length of hospital stay.

## Conclusion

In cases of acute pancreatitis, the maximum CT value of the adrenal glands in the arterial phase of dynamic contrast-enhanced CT was significantly higher than in non-pancreatitis controls, and the intensity of the contrast enhancement correlated with the maximum CRP value and CAR during the subsequent course of pancreatitis. This finding may assist in predicting the course of the acute pancreatitis, especially in patients evaluated as moderate by MCTSI at the time of presentation.

## Data Availability

Data may be available upon reasonable request by contacting the corresponding author.
